# Consequences of the *dynamic triple peak impact factor* in Traumatic Brain Injury as Measured with Numerical Simulation

**DOI:** 10.3389/fneur.2013.00023

**Published:** 2013-03-12

**Authors:** Hans von Holst, Xiaogai Li

**Affiliations:** ^1^Section of Neurosurgery, Division of Clinical Neuroscience, Karolinska InstitutetStockholm, Sweden; ^2^Division of Neuronic Engineering, School of Technology and Health, Royal Institute of Technology (KTH)Stockholm, Sweden

**Keywords:** traumatic brain injury, kinetic energy transfer, finite element modeling, intracranial pressure, strain energy density and first principal strain, dynamic triple peak impact

## Abstract

There is a lack of knowledge about the direct neuromechanical consequences in traumatic brain injury (TBI) at the scene of accident. In this study we use a finite element model of the human head to study the dynamic response of the brain during the first milliseconds after the impact with velocities of 10, 6, and 2 meters/second (m/s), respectively. The numerical simulation was focused on the external kinetic energy transfer, intracranial pressure (ICP), strain energy density and first principal strain level, and their respective impacts to the brain tissue. We show that the oblique impacts of 10 and 6 m/s resulted in substantial high peaks for the ICP, strain energy density, and first principal strain levels, however, with different patterns and time frames. Also, the 2 m/s impact showed almost no increase in the above mentioned investigated parameters. More importantly, we show that there clearly exists a dynamic triple peak impact factor to the brain tissue immediately after the impact regardless of injury severity associated with different impact velocities. The dynamic triple peak impacts occurred in a sequential manner first showing strain energy density and ICP and then followed by first principal strain. This should open up a new dimension to better understand the complex mechanisms underlying TBI. Thus, it is suggested that the combination of the dynamic triple peak impacts to the brain tissue may interfere with the cerebral metabolism relative to the impact severity thereby having the potential to differentiate between severe and moderate TBI from mild TBI.

## Introduction

During the last three decades there has been a substantial increased experience and knowledge about the consequences of traumatic brain injury (TBI) with regard to the neurochemical cascades of events following the kinetic energy transfer into the brain tissue tissue (Narayan et al., 1995). However, there is a lack of knowledge when it comes to the neuromechanical pattern and its consequences.

The awareness of primary prevention of TBI in all categories has improved among the general population resulting in a reduced number of TBI. The same holds true also for the more specific prevention of secondary injuries in the acute neurosurgical treatment. However, despite the numerous publications there is still a lot to be desired when it comes to the neuromechanical cascades immediately after TBI at the scene of accident where the experience is limited. An increased knowledge about the immediate mechanical consequences within milliseconds should have a strong potential to further improve not only primary but also the secondary prevention at the neurosurgical treatment.

The improved computer capacity and the introduction of the neuroengineering field makes it possible to use advanced numerical simulations aiming at better understanding the consequences of TBI and which has been a topic under many previous investigations (Ward et al., 1980, 1997; Anderson et al., 1999; Willinger and Baumgartner, 2003; Zhang et al., 2004; Viano et al., 2005; Balestreri et al., 2006; Kleiven, 2007; Nyein et al., 2010). Various injury predictors have been proposed from numerical modeling or *in vivo* experiments. The first principal strain has been shown to correlate closely with diffuse axonal injuries (Bain and Meaney, 2000; Viano et al., 2005) as well as hemorrhage (Kleiven, 2007). The pressure was suggested to be associated with concussion (Ward et al., 1980) while strain energy density was found to correlate with contusion (Kleiven, 2007). Other predictors include, however not limited to global total energy (Willinger and Baumgartner, 2003) and shear stress (Anderson et al., 1999; Zhang et al., 2004). Thus, assuming that the human brain model is well validated the numerical analysis has an outstanding potential to further improve the existing knowledge about the direct neuromechanical consequences during the following milliseconds after the accident (von Holst and Li, 2013) and also when the patient is under neurosurgical treatment (von Holst et al., 2012). The present study proposes that the immediate consequence of an impact should be looked upon as an effect specifically from the three parameters kinetic energy, intracranial pressure (ICP), and strain level in a sequential manner thereby influencing the brain tissue at different times following the accident.

Thus, the aim of the present study was

– to evaluate the immediate consequences of kinetic energy transfer to the brain tissue focusing on the ICP, strain energy density, and first principal strain level by using the numerical simulation of finite element (FE) model and
– to estimate the kinetic energy impact level which could potentially separate the severe and moderate TBI from mild TBI focusing on the ICP, strain energy density, and first principal strain level.

## Materials and Methods

### The finite element head model

A previously developed and validated numerical model of the human head was used and which includes the scalp, skull, brain, the meninges, and cerebrospinal fluid (CSF) (Kleiven, 2002). A simplified neck, including the extension of the brain stem to the spinal cord and dura mater was also modeled. To cope with large elastic deformations, a hyper-viscoelastic constitutive law was used for the brain tissue. The model has been validated against ICP and brain motion, more detailed information about the model and validations can be found in previous publications (Kleiven and von Holst, 2002; Kleiven, 2006, 2007). The skull bone was modeled as a three-layered structure including inner and outer compact bone and porous bone. During an impact loading, the mechanical parameters of the skull bone are expected to have a strong influence on the dynamic response of the brain. The choice of skull bone parameters in this study was consistent with a previous study (Fahlstedt et al., 2012) and was found to be justified.

### Simulation of impact

**Figure 1:**
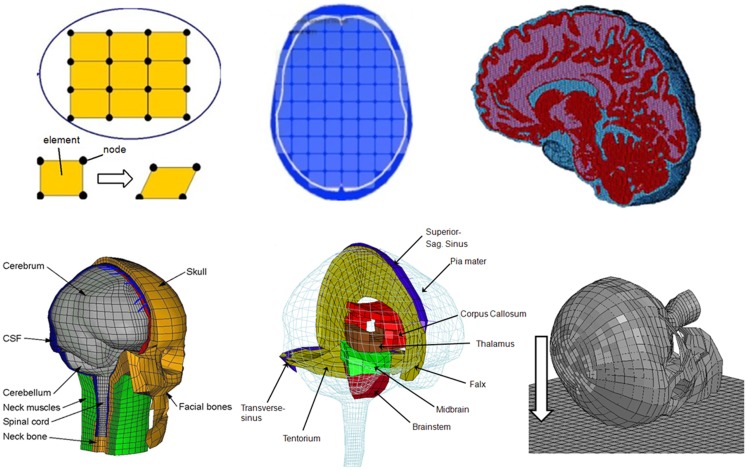
**Schematic development of a FE model (upper row)**. The pictures illustrate the concept of FE model development procedure. First, a continuum is discredited by a number of finite elements connected at their common nodes. The picture in the middle shows an axial slice with elements included, and the picture on the right shows an FE model with detailed anatomical structures. The lower row shows the FE model used in this study (left two), and on the right picture indicates the direction of oblique or rotational impacts to the right frontal lobe of the head which was simulated in this study.

The loading conditions of the impact were based on a real accident to the right frontal lobe in a TBI patient due to a fall. A Computer Tomography (CT) scan on admission to the hospital showed a 3 cm large subdural hematoma on the right side including subarachnoid hemorrhage and multiple contusions in frontal, parietal, and temporal lobes from the medical record, swelling was observed at the right eye and this information was used to estimate the angle and area of impact in the model (Figure 1, lower row). The head model was placed at a very small distance from the impact surface. With a starting point from a non-injured simulation condition the model simulated three falling accidents with an initial velocity of 2, 6, and 10 meters/second (m/s), respectively, to represent minor, medium, and more severe injuries. The external kinetic energy levels were calculated to 227.3, 81.8, and 9.1 J for the 10, 6, and 2 m/s impacts, respectively according to the kinetic energy theorem *E* = 1/2*m*v^2^ where *E* is kinetic energy, *m* is the mass of the head model, and *v* is the initial velocity. The impact ground was simulated as concrete with a Young’s modulus of 30 GPa.

### Estimation of the ICP and strain level

In reality, brain tissue can be seen as a continuum consisting of an infinite number of tissue points. According to the FE theory, FE modeling is a powerful tool to estimate the complex mechanical response of a continuum by dividing it into a finite number of elements (Belytschko et al., 2000). The results obtained at an element are a reasonable approximation to the brain tissue represented by that element. In total the FE head model used in this study consists of around 24,000 elements.

At the time of the simulated impact to the head ICP is defined as the hydrostatic pressure obtained in each of the FEs. For one-dimensional problem, the strain level represents the relative change in length and can be defined as (*L* − *L*_0_)/*L*_0_, where *L*_0_ is the original length and *L* is the length after stretching. For three-dimensional situation, however, a scalar is not enough to represent the complex deformation pattern. Instead a tensor field is needed (i.e., strain tensor) to describe the deformation state. Based on the strain tensor, various scalar indexes can be derived, of which the first principal strain represents the maximum stretching at a tissue point.

When the head is subjected to an impact, the external kinetic energy transferred to the brain causes deformation of nervous tissue and stores the energy in the form of elastic strain energy. This is then translated all the way down to the protein structures at the molecular scale. From the FE model, the kinetic energy transferred to the whole brain can be obtained in the form of strain energy density defined as the strain energy per unit volume of the brain tissue.

## Results

**Figure 2:**
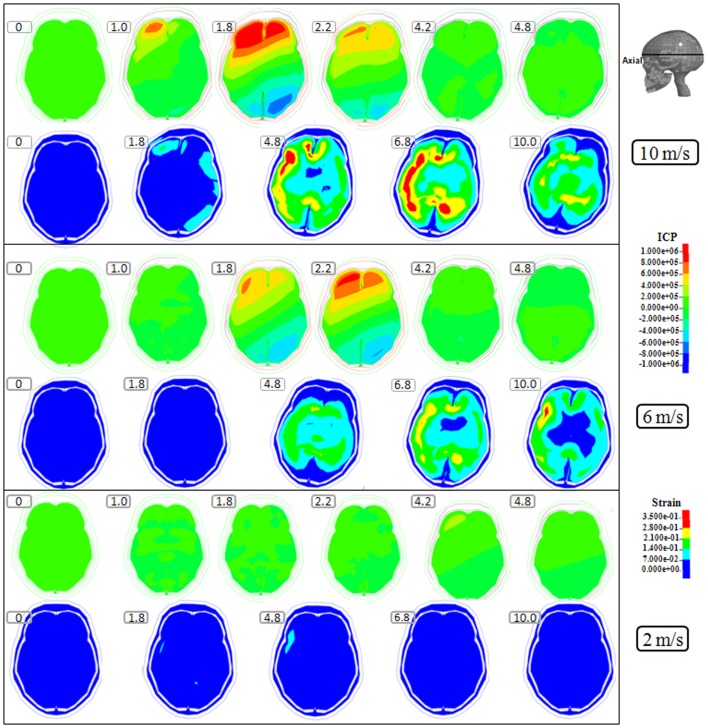
**Representative axial slice of ICP and first principal strain captured at different time points during the impact for velocities of 10 m/s (two upper rows), 6 m/s (two middle rows), and 2 m/s (two lower rows)**. The location of the axial slice is indicated in the figure. The numbers included in the figure represent the time after the impact with unit of millisecond.

**Figure 3:**
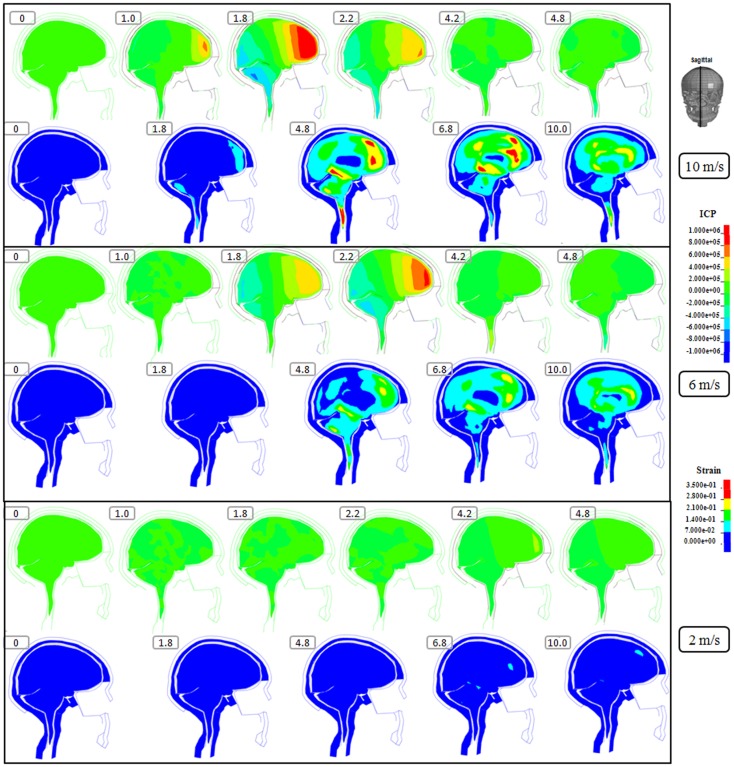
**Representative sagittal slice of ICP and first principal strain captured at different time points during the impact for velocity of 10 m/s (two upper rows), 6 m/s (two middle rows), and 2 m/s (two lower rows)**. The location of the sagittal slice is indicated in the figure. The numbers included in the figure represent the time after the impact with unit of millisecond.

**Figure 4:**
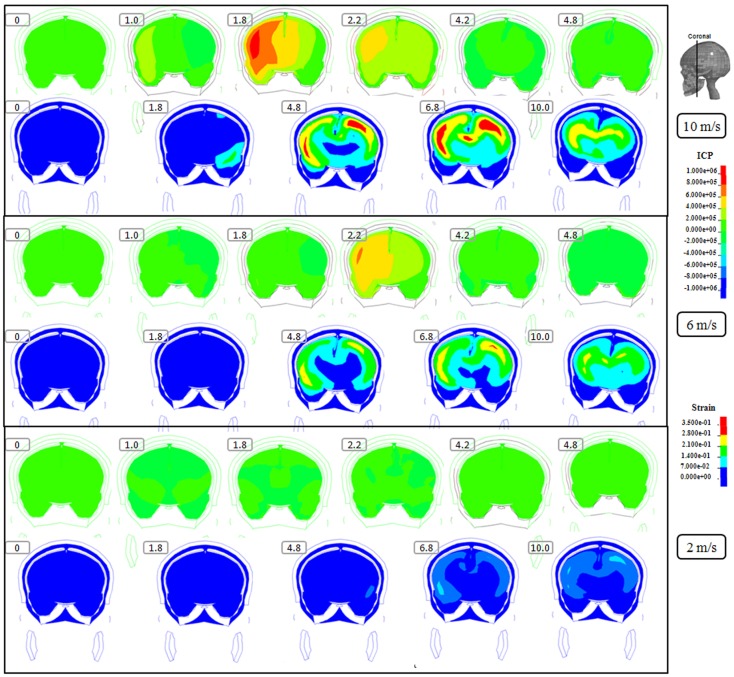
**Representative coronal slice of ICP and first principal strain captured at different time points during the impact for velocity of 10 m/s (two upper rows), 6 m/s (two middle rows), and 2 m/s (two lower rows)**. The location of the coronal slice is indicated in the figure. The numbers included in the figure represent the time after the impact with unit of millisecond.

**Figure 5:**
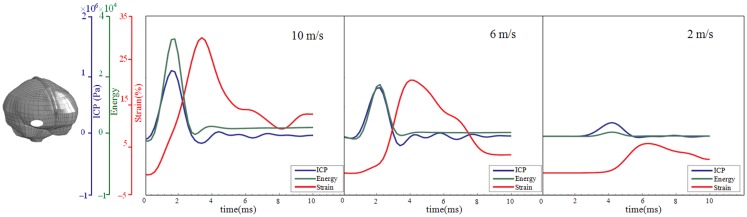
**Time-history curve of ICP, strain energy density, and first principal strain at one element in the right frontal lobe (white area) with the maximum peak ICP value as illustrated in the figure**. *x* Axis represents the time after impact, and *y* axis represents the values of ICP (blue), strain energy density (green), and first principal strain (red). From left to right are the results for the impact velocities of 10, 6, and 2 m/s impact.

The general pattern of the numerical simulation from the three impacts of 10, 6, and 2 m/s was quite different with regard to the head and brain tissue response when comparing the ICP with that of strain energy density and first principal strain as evaluated from the axial, sagittal, and coronal sequences (Figures 2–5). Also, the external kinetic energy levels during the oblique impacts to the head were calculated to 227.3, 81.8, and 9.1 J for the 10, 6, and 2 m/s impacts, respectively.

Following the 10 m/s impact the ICP level was highest in the right frontal lobe at the site of impact area where it reached over 1162 kPa. Then the ICP decreased successively with the distance from the impact site reaching a negative pressure on the opposite side. The patterns of the predicted pressure response within the cranial cavity showed distinct pressure gradients across the entire brain. The pressure varies from a positive pressure close to the impact point to reach a maximum magnitude of negative pressure at the opposite side of the impact which indicated a coup and countercoup pressure phenomenon. The same pattern was found also following the 6 m/s impact, however with a pressure peak reaching about 825 kPa at the impact site. In contrast, the peak pressure following 2 m/s impact reached only 221 kPa at the same area. Moreover, the highest peak pressure came earlier in the two higher impacts. Thus, in the 10 m/s impact the ICP increase started already after 1.0 ms and reached the highest peak at 1.8 ms before normalized after 4.2 ms. The same pattern was true also for the 6 m/s impact although the ICP increase started at 1.8 ms. In contrast, following 2 m/s impact the highest peak pressure of about 400 kPa was found after 4.2 ms (Figures 2–4).

The increased strain levels following 10 m/s impact reached a peak over 35% after 4.8 ms (Figures 2–4, upper rows). For the 6 m/s impact, the strain level in most brain tissue remained lower than 28%, and the peak value was found in time frame of 6.8 and 10.0 ms. The highest strain level occurred later compared to the 10 m/s impact. Following the 2 m/s impact there was a slight and local increase found after 4.8 ms during only a short time (Figures 2–4). Compared to the ICP gradient generated across the brain, the strain level had a different response pattern. Initially, the strain level on the cortical surface and brain stem was high before gradually moving to the central region of the brain especially at the tissue close to falx and tentorium which is stiffer compared to the surrounding brain tissue.

Thus, the kinetic energy transfer resulted in the dynamic triple peak impact consisting of a rapid increased ICP level for a short period while the increased strain level was delayed and more longstanding (Figure 5). To further analyze quantitatively the time frame of the dynamic triple peak impact, the time-history curve for one element at the right frontal brain tissue was plotted which shows that the time for the highest peak values were similar for ICP and strain energy density reaching their peaks in correlation to the impact intensity. The curves were filtered using an SAE 180 low-pass filter. The location of the element in the brain model used for analysis is indicated in the white area (Figure 5, left). All the peaks occur earlier for the more severe injuries due to the higher impact velocities which needs shorter time to reach an impact peak. Of interest is to integrate the strain energy density curve with time thereby representing the total energy density received at this point during the impact. Thus, the results were calculated as 5.81 × 10^4^, 2.76 × 10^4^, and 2.14 × 10^3^ respectively for each of the three impacts. Further, the ICP level had returned to normal when the strain level reached the peak value. As the first principal strain level increase was delayed and prolonged, the brain tissue encountered a dynamic triple peak impacts with the highest impact for 10 m/s followed by the 6 m/s and with the lowest impacts in all categories following 2 m/s (Figure 5).

## Discussion

The present numerical simulation study shows for the first time that the kinetic energy transfer in fact results in a dynamic triple peak impact event during the first milliseconds after the accident to the head. Consequently, this may have a profound effect on the cerebral metabolism. An FE model of the human head has been investigated in numerous studies aiming at better understanding the complex mechanisms of TBI. From the FE model simulations, spatial and temporal distribution of the mechanically response parameters can be determined mainly in terms of ICP, strain energy density, first principal strain level. If one parameter or a combination of several parameters obtained from the FE model is correlated with the site and severity of the injuries, the result can be used to better understand the consequences of the dynamic triple peak impact shown in the present study. Also, such a combined evaluation of several parameters should be used as an injury prediction of TBI consequences. Many predictors have been proposed in the literature based on either numerical simulations or *in vivo* experiments. Among them are ICP (Ward et al., 1980; Kleiven, 2007), strain energy density (Kleiven, 2007), first principal strain (Viano et al., 2005; Kleiven, 2007), global total energy (Willinger and Baumgartner, 2003), and shear stress (Anderson et al., 1999; Zhang et al., 2004). In this study, we focused on the three widely used parameters ICP, strain energy density, and first principal strain.

The kinetic energy transfer of 227.3, 81.8, and 9.1 J defined for the three impact scenarios in this study should have a profound effect on the cerebral metabolism. Earlier analytical and animal studies proposed a peak ICP threshold of 235 kPa for fatal or serious head injuries and 173 kPa for moderate head injury (Ward et al., 1980). From a clinical perspective it has been shown that high ICP is strongly associated with fatal outcome in patients with TBI (Balestreri et al., 2006). Thus, in contrast to clinically increased ICP, which is developed at a static or semi-static event, our simulation shows a substantially increased ICP developed during dynamic impacts and lasting for several milliseconds. The pressure reached a maximum positive value at the impact site while a maximum magnitude of negative pressure at the opposite side of the impact which indicated a coup and countercoup pressure phenomenon. Although countercoup injury is widely recognized both in clinics and experiments, the mechanisms is still controversial nowadays (Post and Hoshizaki, 2012). Also, the difference in increased ICP is substantial between 10 and 6 m/s impacts compared to that of 2 m/s impacts. Based on our results it is tentative to suggest a possible separation of severe and moderate TBI following 10 and 6 m/s impact from that of mild TBI following 2 m/s impacts. The increased ICP developed during dynamic events may very well influence the cerebral metabolism in such a way that it makes the brain tissue more sensitive to the secondary increased ICP often found during the clinical treatment of TBI.

It has been suggested that a threshold of strain energy density at 2.1 kJ/m^3^ indicated a 50% chance of concussion similar to that found in a range of about 0.8–1.9 kJ/m^3^ (Ward et al., 1997). In the present study the strain energy density reached a peak value of about 33 and 18 kJ/m^3^ following 10 and 6 m/s impacts, respectively, while the 2 m/s impact reached a peak of only 2 kJ/m^3^. In line with our suggestion from the dynamically increased ICP also the strain energy density may stress the cerebral metabolism in the deformed brain tissue which is then more sensitive to the secondary injuries developed within the nearest days after the impact. Also, the calculated area under the strain energy density curve may support clarify the dysfunction of the cerebral metabolism in future experiments.

An increased first principal strain level of 21% has been shown to result in a 50% probability to cause mild TBI (Kleiven, 2007). This is similar to the values demonstrated from *in vivo* axonal stretching experiments that a strain level of approximately 21% will elicit electrophysiological changes while a strain level of approximately 34% will cause morphological signs of damage to the white matter (Bain and Meaney, 2000). In our present study the first principal strain level increased to over 30 and 20% for 10 and 6 m/s impacts, respectively. This may very well result in an electrophysiological dysfunction which may jeopardize the cerebral metabolism in the brain tissue both at the time of impact but also later on.

The kinetic energy transfer of 227.3, 81.8, and 9.1 J defined for the three velocity impact scenarios in this study should be highlighted as this may be beyond the energy forces that can be tolerated in some of the normal cellular metabolism. Since the nervous tissue contains a substantial amount of protein structures, it is of clinical interest to evaluate whether or not the kinetic energy transferred to brain tissue during an impact has the potential to unfold the native proteins. As suggested from earlier experimental data a high pressure may induce unfolding of the protein structures as the pressure exerts a destabilizing effect on the folded protein structures (Hummer et al., 1998; Hillson et al., 1999). Upon unfolding the protein structures developed a swelling process as has been shown in a previous experimental study (Jha et al., 2009). As shown from our previous studies (von Holst and Li, 2013), when severe enough, the external kinetic energy may unfold native proteins after severe and moderate TBI. The unfolded protein structures with a larger number of hydrophilic amino acids residues exposed to the aqueous environment within the nervous tissue tend to attract more water molecules and, hence, initiating the cytotoxic brain tissue edema often seen in TBI patients.

The triple peak impact is illustrated for one element at the frontal lobe close to the impact site elements at other parts of the brain is expected to have also experienced an impact peak sequential, however, may have different magnitude ratios at different locations of the brain. Disturbance of cerebral metabolism following TBI have been reported in both animal models and in clinical measurements including but not limited to elevated extracellular glutamate and lactate and decreased glucose (Alves et al., 2005), increased glycolysis and or/decreased oxidative metabolism (Casey et al., 2008). Thus, these provide clear evidence that there is a metabolic disturbance following TBI. However, the exact mechanisms how each of the impact peaks affects the cerebral metabolisms remain unclear and they need further experimental investigations.

An accurate reconstruction of real accident demands information regarding the accident impact ground material properties, impact velocity and angle etc. Due to the lack of the information, the impact location and angle in the model was estimated from the medical image where swelling was observed at the right frontal lobe. The impact ground is assumed to be with similar stiffness as concrete. However, in reality the impact ground may be different. Therefore, the simulation results presented in this study should be interpreted with caution since the conclusions are limited by the specific modeling conditions and impact parameters. Nevertheless, the dynamic triple peak during an impact presented in this study is expected to remain valid for slightly different global mechanical loads. Future investigations could include a larger number of trauma patients with accident report at the scene of accident and their medical reports. From numerical simulations the intracranial response during various loading conditions could be predicted and this should provide more insight into the relative importance of different impact peaks in a certain type of brain injury.

There has been a substantial interest to better clarify the precise mechanisms of brain injury aiming at improving the clinical treatment of patients with TBI. However, this has not yet been fully established (Yang et al., 2011; Post and Hoshizaki, 2012). It has been widely recognized that there are limitations with using only single parameters related to TBI. In this study, rather than suggesting another predictor, we propose that the external kinetic energy transferred to the head during an accident may result in at least three sequential impacts to the brain tissue defined as the immediate dynamic triple peak impact factor which occurred in a sequential manner first showing strain energy density and ICP and then followed by first principal strain. The denominator in common for the three parameters is the impact it has on influencing different cerebral metabolic situations after the accident. Even if it will not alter the cerebral metabolism at the time of accident, it is tentatively suggested that the dynamic triple peak impact factor influences the cerebral metabolism in such a way that it is more sensitive to the neurochemical cascades often found after a short time in patient with severe and moderate TBI. Thus, it is evident that the immediate and dynamic triple peak impact factor presented in this study can be looked upon as a forerunner thereby making the cerebral metabolism in the injured area more vulnerable to secondary dysfunctions.

## Conflict of Interest Statement

The authors declare that the research was conducted in the absence of any commercial or financial relationships that could be construed as a potential conflict of interest.
